# Signaling with Homeoprotein Transcription Factors in Development and Throughout Adulthood

**DOI:** 10.2174/1389202911314060009

**Published:** 2013-09

**Authors:** A Prochiantz

**Affiliations:** College de France, Centre for Interdisciplinary Research in Biology (CIRB), UMR CNRS 7241/INSERM 1050, Labex Memolife, PSL Research University, Development and Neuropharmacology group, 11 place Marcelin Berthelot, 75231 Paris Cedex 05, France

**Keywords:** Homeoproteins, Signaling, Development, Morphogenesis, Nervous System, Plasticity.

## Abstract

The concept of homeoprotein transduction as a novel signaling pathway has dramatically evolved since it was first proposed in 1991. It is now well established in several biological systems from plants to mammals. In this review, the different steps that have led to this unexpected observation are recalled and the developmental and physiological models that have allowed us (and a few others) to consolidate the original hypothesis are described. Because homeoprotein signaling is active in plants and animals it is proposed that it has predated the separation between animals and plants and is thus very ancient. This may explain why the basic phenomenon of homeoprotein transduction is so minimalist, requiring no specific receptors or transduction pathways beside those offered by mitochondria, organelles present in all eukaryotic cells. Indeed complexity has been added in the course of evolution and the conservation of homeoprotein transduction is discussed in the context of its synergy with *bona fide* signaling mechanism that may have added robustness to this primitive cell communication device. The same synergy possibly explains why homeoprotein signaling is important both in embryonic development and in adult functions fulfilled by signaling entities (e.g. growth factors) themselves active throughout development and in the adult. The cell biological mechanism of homeoprotein transfer is also discussed. Although it is clear that many questions are still in want of precise answers, it appears that the sequences responsible both for secretion and internalization are in the DNA-binding domain and very highly conserved among most homeoproteins. On this basis, it is proposed that this signaling pathway is likely to imply as many as 200 proteins that participate in a myriad of developmental and physiological pathways.

## SERENDIPITY

**Fig. (1):**
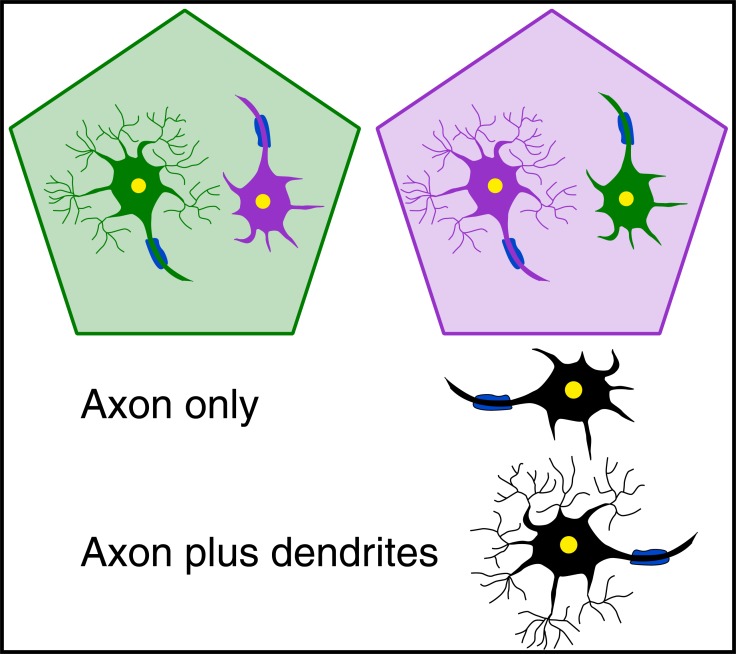
Regulation of neuronal polarity through region-specific neuro-glial interactions. Neurons cultured on astrocytes from the same brain "compartment" (green on green or pink on pink) develop a dendritic tree more profuse than neurons grown on astrocytes from a different "compartment" (green on pink or pink on green).

The identification of a novel signaling pathway based on homeoprotein (HP) transduction finds its origin in the observation that neurons and astrocytes (a class of non-neuronal cells) isolated from different brain regions maintain their regional properties. This was deduced from the finding that neurons grown on astrocytes from the same region (homotopic co-cultures) develop a profuse dendritic arbor very different from the one they would develop in heterotopic co-cultures [1, 2] (Fig. **1**). In 1984, this observation was strikingly reminiscent of the recently discovered homeogenes in the sense that a connection could be made between shape and position [3]. This is why we speculated that the same class of genes, homeogenes, encoding positional information, may regulate the shape of single cells in addition to that of organs. This seemed sensible since many of the molecules able of modifying cell and organ shapes might be of similar nature (adhesion molecules, cytoskeleton) and thus the putative (at the time elusive) targets of homeogene-encoded HP transcription factors.

The DNA-binding domain of many HPs, called the homeodomain (HD), is highly conserved among a large number of HPs [4, 5]. On this basis we speculated (and later verified [6]) that injecting a HD within neurons may chase the endogenous HPs from their cognate binding sites, thus changing neuronal shape if our hypothesis was confirmed. Consequently, we produced the HD of the Drosophila Antennapedia genes (AntpHD) and introduced it into nerve cells by scrape loading with the expected results of a change in neuronal morphology [7]. Because the scrape-loading protocol results in a lot of material being in the culture medium, we did a control experiment consisting simply in adding the HD to the culture medium. To our surprise we observed the same changes in neuronal phenotypes, making it likely that we were dealing with an artifact. However, before giving up we labeled the HD with a fluorescent tag and this is how we found that AntpHD was internalized by live cells and addressed directly to their cytoplasm and nucleus [7] leading to morphological changes [8, 9].

## CELL BIOLOGICAL STUDIES

Although this seems now obvious, twenty years ago the idea that HPs may regulate the shape of single cells was troubling. Even more troubling was the observation that a 60 amino-acid long polypeptide could travel across biological membranes and gain direct access to the cytoplasm, a fact at odds with the Berlin wall concept of the plasma membrane. Indeed the perspective that this may be a shared property of many HDs (because of their conserved structure) and, beyond, of full-length HPs was difficult to consider with complete serenity. The latter doubts were also shared by our own group, leading us to develop a cell biological study of HD internalization and later of HP intercellular transfer [10-13]. I do not intend to go into the details of these studies that have been recently reviewed [14-17] but to make a few comments on this novel signaling pathway. 

**Fig. (2):**
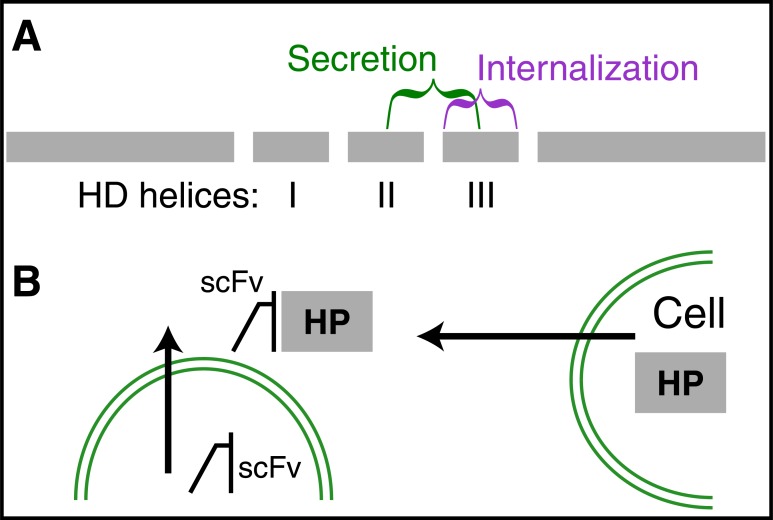
The single-chain antibody strategy to neutralize extracellular HPs. Single-chain antibodies (scFvs) are encoded by minigenes resulting from the cloning of the light and heavy variable chains linked with a hinge sequence and preceded by a secretion signal peptide. They are secreted into the extracellular space where they neutralize secreted HPs.

A first comment is that HP transfer is permitted by the presence, within the HD of secretion and internalization signal peptides highly conserved within HPs [15]. This is particularly so of the internalization sequence that corresponds exactly to the third helix of the HD [18, 19]. This conservation explains that 10 out of 10 tested HPs translocate between cells and strongly suggests that this is also the case for most of the 200 (approximately) HPs that have been identified. This presence of the two key transfer sequences in the DNA-binding domain has for consequence that it is impossible to mutate them without modifying HP cell-autonomous transcriptional activity. This is why we developed the single-chain antibody strategy based on the *in vivo* expression of a secreted antibody allowing one to neutralize the extracellular HP while leaving untouched its cell autonomous activities (Fig. **2**). A second comment concerns the translocation of the third helix of the HD, known as Penetratin, and that of full-length proteins. The punctual mutations that block Penetratin internalization also block that of full-length HPs suggesting that the two events are related. However, it is not impossible that other sequences are required for HP internalization and also for the specific recognition of target cells as will be developed later. Finally it is quite interesting that secretion and internalization do not use the same signal peptides. In particular the group of Alain Joliot has shown that internalization and secretion are distinct phenomena and that the check-in pathway differs totally from the check-out one [20].

## IS HP SIGNALING A VERY ANCIENT PHENOMENON?

**Fig. (3):**
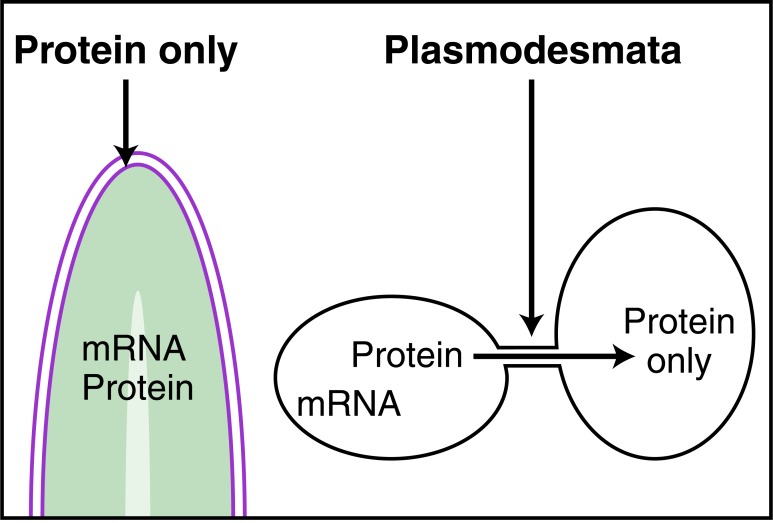
Plasmodesmata for protein transfer and signaling in plants. In plants the presence of cellulose walls explains the signaling function of cytoplasmic intercellular bridges called plasmodesmata. Plasmodesmata allow the passage of several signaling entities, including proteins.

A fascinating study achieved by Joliot and collaborators concerns the similarities between plant and animal HD transduction [21]. The intercellular transport of proteins, including HPs, is a well-known phenomenon in plants. This is normally explained by the fact that plants, because their cells are separated by cellulose walls, use intercellular corridors, called plasmodesmata, allowing intercellular exchanges, including that of proteins [22]. Still in plants, it was shown that the HD is necessary and sufficient for HP transfer and mutations were identified that impair the latter transfer [23, 24]. In a key experiment Joliot and colleagues have shown that the HD of Knotted-1 (KN1) a Meis-type plant HP that moves from the core mesenchyme to the epithelial layer of the shoot meristem (Fig. **3**) is also transported between animal cells, therefore in absence of physical contacts between the cells. Moreover the KNM6 mutant that does not transfer in plants does not travel between animal cells and a revertant that transfers again in plants, transfers as well in animal cells. This series of experiments demonstrate that KN1 HD does not need plasmodesmata for transfer and strongly suggests that HP signaling has preceded the separation between metaphytes and metazoans. If so, it may represent a very ancient mode of signal transduction present in the first pluricellular organisms and possibly active in unicellular organisms.

**Fig. (4):**
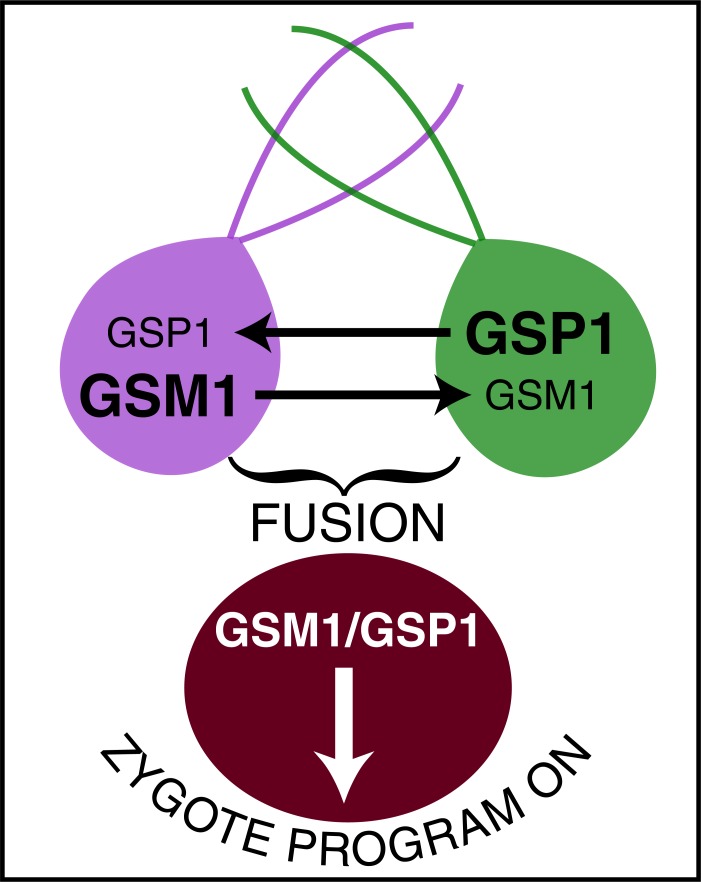
Signaling with HPs in unicellular green algae conjugation: a hypothesis. The mating types in *Chlamidomonas rheinardi* are defined by two HPs, GSM1 and GSP1. A classical view is that following fusion the two HPs form heterodimers and trans-activate zygotic genes. The proposal is that fusion between the gametes is posterior to HP exchange and takes place only if the two gametes are of opposite signs.

To my knowledge, this hypothesis has not been addressed, but is may be interesting to consider it in the context of unicellular conjugation. *Chlamidomonas rheinardi* a unicellular green alga multiplies as haploid mt+ and mt- "gametes" that conjugate under food deprivation to produce dormant zygotes that will enter meiotic divisions when food returns. The present view is that the gametes pair and fuse thanks to adhesion molecules expressed at the surface of the flagella and that following fusion, two HPs GSM1 and GSP1 respectively expressed by the + and - haplotypes form a complex that initiates the transcription of zygotic genes [25] (Fig. **4**). Based on sequence analysis and on the belonging of at least one of the HPs (GSM1) to a class of HPs known to translocate (KN1 family), it can be proposed that fusion takes place following HP transport if and only if the two gametes are of different haplotypes. This is indeed quite parsimonious as it would prevent fusion between gametes of the same "gender", an unlikely way to give rise to a progeny.

Indeed this raises the issue of a possible link between the "invention" of pluricellularity, a highly debated issue, and conjugation. It is not in my intention to develop this hypothesis further, but I will point out that HPs can be found in Metazoa, Fungi, Plantae, Amoebozoa, Heterokonta and Trichomonas [26]. It is thus obvious that they have appeared before most classical signaling molecules. If we see HP transfer in such an evolutionary perspective, it can be speculated that the recruitment of classical signaling pathways may have added robustness to this early HP-dependent signaling strategy. In that case one understands better the interaction between HP signaling and other signaling pathways that we have demonstrated at least for three HPs, as discussed below.

## HP SIGNALING COOPERATES WITH CLASSICAL SIGNALING PATHWAYS

**Fig. (5):**
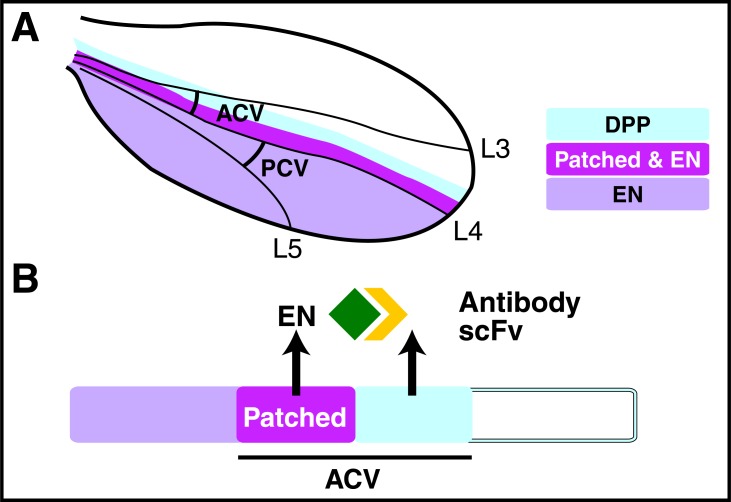
Extracellular Engrailed is a morphogen for anterior cross vein formation in the Drosophila wing disk. The anterior cross vein (ACV) crosses the Patched domain (where Engrailed is expressed) and the DPP domain (where Engrailed is not expressed). A single chain antibody against Engrailed (scFv, yellow) secreted by the DPP domain neutralizes Engrailed (EN, green) produced by the Patched domain. As a result the ACV is not formed, demonstrating an extracellular morphogenetic function of Engrailed.

This interaction concept will be illustrated in the case of the morphogenesis of the anterior cross vein (ACV) in the fly wing disk. In this disk Engrailed (En) HP is only expressed in the posterior compartment with the exception of the Patch domain (Fig. **5**). The ACV is in the anterior domain, half of it in the Patch domain and the other half in a more anterior domain including the domain of DPP expression. In a recent study we demonstrated that, in contrast with the posterior cross vein (PCV) which requires the cell autonomous expression of En for its development, ACV formation requires the secretion of En by the Patch domain [27]. This demonstration is based on the expression under the control of different promoters of an En-targeting RNAi construct and of an extracellular single chain antibody directed against En (saEn). The results schematized in (Fig. **5**) demonstrate that En secreted by the Patch domain is necessary for ACV formation and acts in a non-cell autonomous way. Expressing the secreted antibody in the DPP domain (where En is not expressed) traps extracellular En "en route" from the Patched domain, resulting in a missing ACV. It is known that DPP induces ACV formation [28] and it was necessary to reconcile the two pathways. DPP signaling leads to the phosphorylation of Mother Against DPP (MAD) and we found that MAD is not phosphorylated in absence of extracellular En, thus that full DPP signaling requires En secretion [27]. We have not deciphered the mechanism in detail but could verify that embryos heterozygote for En and DPP do not form an ACV, genetically confirming an interaction between the En and DPP signaling pathways [27].

**Fig. (6):**
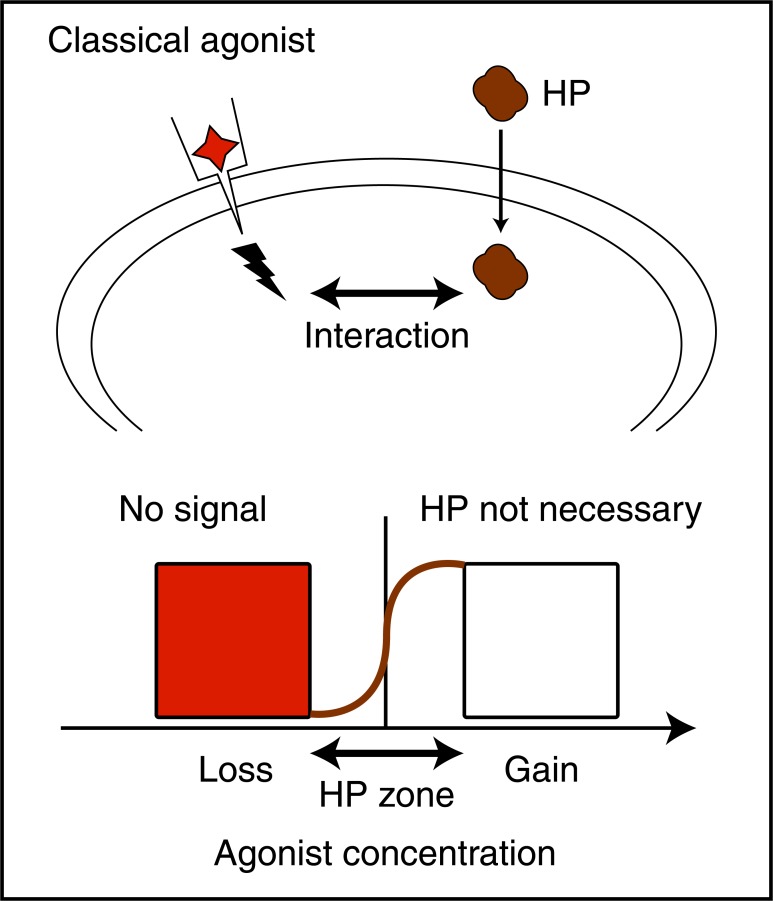
Synergy between HP and bona fide signalling. The finding illustrated here (see text for references) is that HP signaling synergizes with classical signaling factors expressed at physiological concentrations. In absence or gain of function of classical signaling HP signaling is useless.

Interactions between HP signaling and classical signaling pathways were also found in vertebrates in the case of axon guidance by extracellular Engrailed1 (En1) and Engrailed2 (En2) (interaction with EphrinA5 signaling) [29] and the regulation of oligodendrocyte precursor (OPC) migration by Pax6 (interaction with Netrin signaling) [30]. The scheme of (Fig. **6**) describes our present understanding of these interactions. Seemingly, HP signaling by itself is insufficient in absence of an associated growth factor or morphogen (loss of function). Conversely, it becomes unnecessary when the associated classical signaling pathway is over-active (massive gain of function). This implies that HP transduction works at physiological concentrations of the associated factor by enhancing its activity. Because most experiments done *in vitro* or *in vivo* are based on loss and gain of function it is not surprising that HP signaling has escaped the attention of many investigators.

## AXON GUIDANCE

**Fig. (7):**
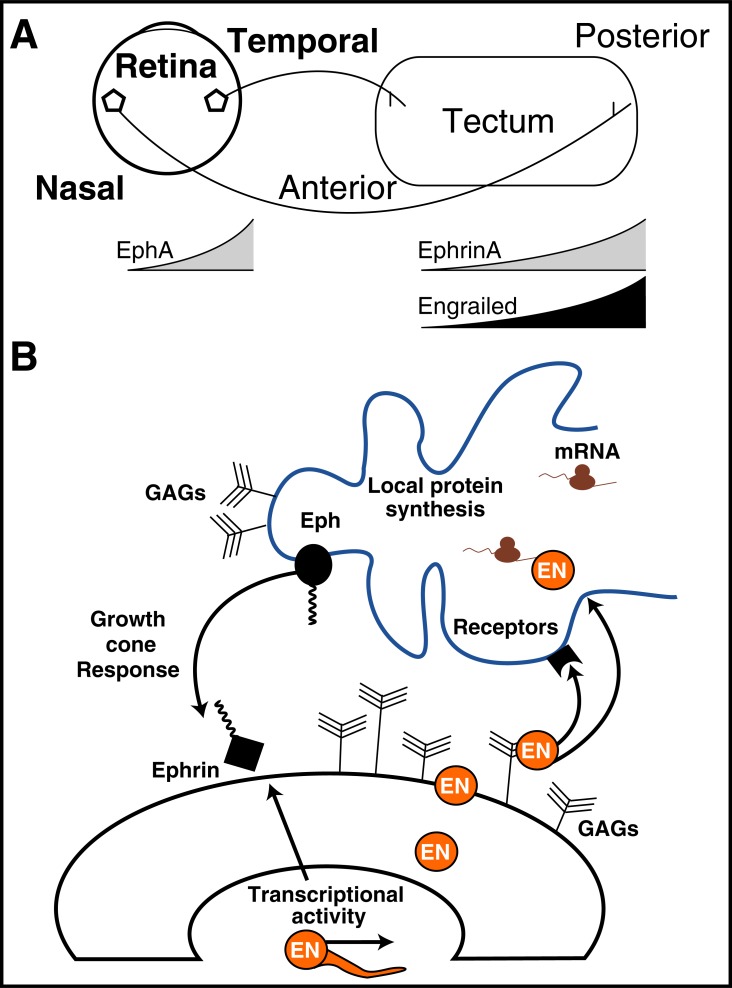
Engrailed guiding activity for Retinal Ganglion Cells. **A**. Scheme of the retina/tectum patterning illustrating the projection of the nasal-temporal axis of the retina on the posterior-anterior axis of the tectum. In the tectum EphrinA5 shows an anterior/low and posterior/ high graded expression due to the same graded expression of Engrailed (En1 and En2) that regulates EphrinA5 expression. In the retina EphA2 (a EphrinA5 receptor) shows a graded expression (nasal/low and temporal/high) that is preserved at the level of growth cones. **B**. Scheme of the role of En1/2 as a guidance factor for RGC axons. En1/2 in the tectum regulates EphrinA5 expression (cell autonomous activity). It is also secreted (graded extracellular expression) and transfers into the growth cones (blue line) where it regulates the translation of factors necessary for EphrinA5/EphA2 signaling to take place.

Axon guidance, as all guidance phenomena, relies on the ability of the moving entity, here the growth cone, to read directional cues expressed within reach of its filopodia [31-33]. The cues, attractive or repulsive, are often expressed in gradients, and the most popular example of such a graded expression is provided by EphrinA5 with its anterior (A)/low: posterior (P) /high expression along the AP axis of the superior colliculus (Sc)/tectum [34]. Because there is a corresponding nasal-temporal graded expression of EphA2 (an EphrinA5 receptor) in the retinal ganglion cells (RGCs) and at the levels of their growth cones, this leads to the projection of the Temporal-Nasal axis of the retina onto the AP axis of the tectum (Fig. **7**). Indeed above a certain level of EphrinA5/EphA activity, the axons stop progressing due to RhoA activation and the ensuing growth cone collapse [35].

The graded expression of EphrinA5 in the tectum is under the control of the graded expression of En1 and En2 (collectively En1/2), leading us to speculate that En1/2 may participate in axon guidance. (Fig. **7**) summarizes our recently published finding that En1/2 shows a graded A-low: P-high expression at the surface of the tectum. Neutralizing extracellular En1/2 with electroporated saEN leads to the misguidance of temporal axons that aberrantly migrate onto the posterior part of the tectum, confirming the role of this HP in guidance [29]. Studies* in vitro* demonstrate that the latter axon guidance requires En1/2 internalization and the local translation of several mRNAs with no immediate necessity for transcriptional activity [36].

In the context of reconciling En1/2 and EphrinA5 signaling, we analyzed the response of temporal growth cones to increasing concentrations of EphrinA5 and determined a sub threshold concentration at which no repulsion or collapse was measurable [36]. Adding inefficient nM amounts of En1/2 to EphrinA5 at sub threshold concentrations reactivated repulsion, reinforcing the cooperation concept proposed in (Fig. **6**). Interestingly, this cooperation requires En1/2 internalization (not observed with an internalization mutant) and local protein synthesis (not observed with protein synthesis inhibitors and not affected by transcription inhibitors) [29].

## LOCAL PROTEIN TRANSLATION

The implication of local protein translation is reminding of Bicoid as a regulator of Caudal mRNA translation in fly development [37-39]. In this system, Bicoid was defined as a morphogen acting non-cell autonomously (the fly is a syncitium at this stage) [40, 41] and regulating translation [37-39]. The ability of HPs to regulate translation seems to be dependent on their association with eIF4E, a translation initiation factor for capped mRNAs [42]. On the basis of the conservation of a eIF4E-binding domain, it has been proposed that over 200 HPs can bind eIF4E, thus regulate translation and we have verified the existence of such an interaction for Emx2, En2 and Otx2 [43].

**Fig. (8):**
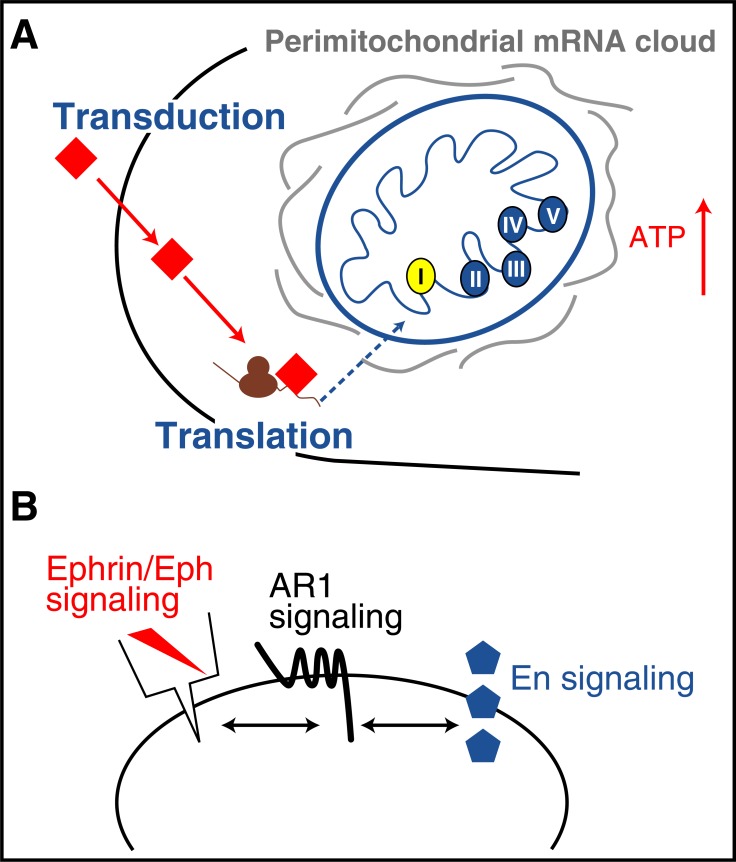
Mitochondrial activity and multi-signaling synergy. **A**. En1/2 internalized by the growth cones regulates the translation of nucleus-encoded mitochondrial mRNAs. The proteins (primarily Ndufs1 and Ndufs3) are imported and induce ATP synthesis. **B**. ATP synthesized following En1/2 regulation of complex 1 activity is secreted and degraded into Adenosine (not illustrated). This allows the stimulation of AR1 Adenosine receptors. It is this stimulation that allows the synergy between En1/2 signaling and EphrinA5/ EphA2 signaling (see text for description and references).

It was important to identify which mRNAs are translated in the growth cones upon En1/2 internalization. To that end RGC growth cones, isolated from E14.5 mouse tectum, were incubated with or without En1 and the protein entering translation identified by isolating polysomes or purifying neo-synthesized proteins for mass spectroscopy. The results of these analyses were extremely striking as over 60% of the targets participate in mitochondrial physiology [44, 45]. This might not be so surprising as most mitochondrial mRNAs are transcribed in the nucleus and anchored at the surface of mitochondria as they travel at diverse points of the cell, including the growth cones and the sub-synaptic compartment. These mRNAs need the eukaryotic protein synthesis apparatus and are thus necessarily translated locally before import into mitochondria (Fig. **8A**).

Among the most highly regulated proteins are Ndufs1 and Ndufs3, two complex I proteins, resulting in ATP synthesis [44]. We found that this newly-synthesized ATP is secreted and degraded into Adenosine resulting in an activation of Adenosine receptor 1 (AR1). The entire process takes less than 100 seconds on average and is necessary for the EphrinA5 response [45]. In fact, blocking AR1 blocks EphrinA5 activity and adding an AR1 agonist permits the collapse of temporal growth cones at sub threshold EphrinA5 concentrations in absence of En1. Taken together, this means that the cooperation between En1 and EphrinA5 requires En1-induced ATP synthesis and AR1 activation (Fig. **8B**).

If we now come back to an evolutionary viewpoint and consider the possibility that HPs were invented before signaling molecules, then a logical conclusion would be that ATP synthesis regulation may have been one of the first function of HPs as translation regulators. This would make the non-cell autonomous regulation of ATP synthesis the first signal transduction mechanism in the evolution of signaling. Needless to say such a regulation of local metabolism may also apply to HP cell autonomous functions, including the regulation of cell motility. 

## REGULATION OF A CRITICAL PERIOD OF PLASTICITY IN THE VISUAL CORTEX

HPs are not only expressed early in development but also postnatally and in the adult. If we think of the non-autonomous activity of En1 on the translation of proteins involved in mitochondrial activity, transfer at the synaptic level may be an important regulator of axon maintenance. The latter possibility has recently been suggested by the finding that En1-regulated Lamin B synthesis allows axon maintenance in the adult frog [46]. In fact, given the high energy levels required to maintain ionic gradients through the Na+/K+ ATPase activity, it is possible that HP transfer in the adult participates in synaptic metabolism and activity. This might be an important issue as many neurological and psychiatric diseases have a strong metabolic facet [47-51]. Before coming back to this point, I will describe a study achieved in collaboration with the group of Takao Hensch that has attracted our attention toward the possibility that HP signaling may allow us to better understand some aspects of brain physiopathology. 

**Fig. (9):**
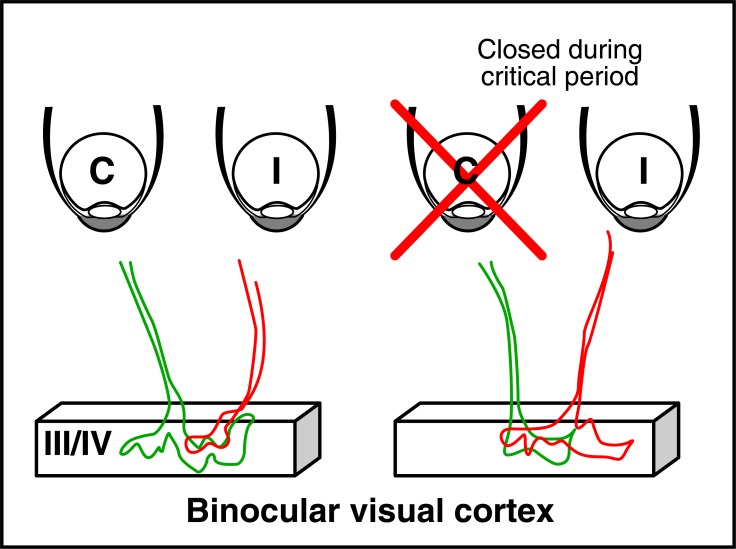
Eye competition in layer 3/4 of the binocular visual cortex. On the left side is illustrated the normal situation when the two eyes are competing during the critical period (CP). In the mouse the contralateral eye (C, green) is dominant and occupies more space than the ipsilateral eye (I, red) in layers 3/4 of the binocular visual cortex. The right side illustrates the shift in ocular dominance when the contralateral eye is closed during CP. This shift does not take place if the eye is closed before CP opening or after CP closure.

**Fig. (10):**
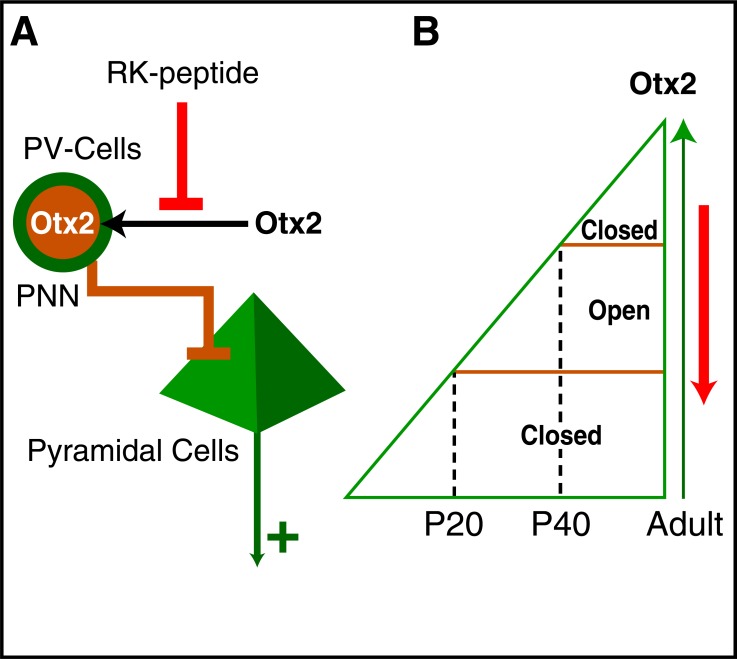
Regulation of the Excitatory/Inhibitory balance by Otx2 transfer. **A**. Otx2 binds PNNs through its GAG-binding domain (RK domain) and is internalized by PV-cells. Before CP opening or during CP, this internalization drives PV-cell maturation and enhances its inhibition strength upon Pyramidal cells, inducing a shift in the excitatory/inhibitory (E/I) balance. Infusing the RK peptide (the Otx2 PNN-binding domain) decreases Otx2 internalization and reopens a period of plasticity. **B**. According to this 2 threshold model, the accumulation of Otx2 (green arrow) opens at P20 (threshold 1) and closes at P40 (threshold 2) a critical period for plasticity. The permanent transfer of Otx2 maintains plasticity in a closed state throughout adulthood. Infusing the RK-peptide (A) blocks Otx2 internalization in the adult and reopens a period of plasticity (red arrow).

During its post-natal development the cerebral cortex goes through a series of critical periods (CPs) during which it adapts to the extracellular environment. Since the pioneering work of Hubel and Wiesel CP for binocular vision has developed as the most studied example of cerebral cortex plasticity [52, 53]. RGCs from binocular retina project onto the dorsal thalamus where relaying thalamic neurons project onto layer 3/4 of the binocular visual cortex (Fig. **9**) to form synapses with a specific class of inhibitory fast-spiking interneurons. These interneurons synthesize parvalbumin (why they are called PV-cells) and are surrounded by glycosaminoglycan (GAG)-rich perineuronal nets (PNNs) that can be specifically decorated by the Wisteria floribunda lectin (WFA) (Fig. **10A**).

In the mouse, PV-cell maturation marked by PV synthesis and PNN assembly takes place between P20 and P40, defining the CP for binocular vision. During this period, closing an eye leads to the withdrawal of the corresponding terminals in layers 3/4 of the binocular visual cortex. These terminals are replaced by terminals from the other eye, leading to an irreversible loss of visual acuity (amblyopia) for the closed eye (Fig. **9**). Closing the eye before of after CP has no effect on visual acuity. PV-cells form synapses on the cell body of pyramidal cells in layer 5 of the cortex and their maturation shifts the excitatory/inhibitory (E/I) balance toward inhibition (Fig. **10A**). 

In the course of our study on Otx2 expression in the cortex we observed that the amount of Otx2 in PV-cells correlates with their maturation (PV expression and WFA staining) [54]. We also found that Otx2 was imported into PV-cells as the Otx2 locus is totally inactive at post-natal stages in the cerebral cortex. This led us to infuse Otx2 into the visual cortex and to show that it is specifically captured by PV-cells and turns on their maturation. This experiment, plus loss of function approaches, allowed us to establish that Otx2 is necessary and sufficient to open CP at P20 and close it at P40.

## REOPENING PLASTICITY IN THE ADULT VISUAL CORTEX

It came as a surprise that Otx2 infused in the visual cortex is specifically captured by PV-cells and excluded from most other cell types [54]. This suggested that PV-cells express a binding site for Otx2 and, conversely, that Otx2 presents a specific domain allowing PV-cell recognition. As already underscored PV-cells are surrounded by PNNs rich in proteoglycans (PGs) and GAGs providing a possible substrate for Otx2 recognition. This hypothesis was verified thanks to the hydrolysis of the GAGs by the infusion of Chondroitinase ABC (ChABC) and the observed consequence of a decreased accumulation of endogenous Otx2 by PV-cells [55]. This led us to identify within Otx2 a GAG-binding domain which is necessary for the recognition of PV-cells by this HP. This sequence RKQRRERTTFTRAQL was mutated into AAQRRERTTFTRAQL leading to a loss in internalization specificity and the uptake of Otx2 by diverse cell types in addition to PV-cells. As another HP (En2) is not specifically captured by PV-cells, this raises the possibility of a sugar code for HP recognition [55].

The affinity of the RK peptide for disulfated Chondroitine Sulfates (CS-D and CS-E) is in the nM range, suggesting that it could be used as an antagonist for the *in vivo* capture of endogenous Otx2 by PV cells. This is indeed the case and in contrast with the AA-peptide or an RK-scrambled peptide, the infused RK-peptide blocks endogenous Otx2 capture by PV-cells [55] (Fig. **10A**). Accordingly, it was reported that mutating sulfotransferases responsible for the synthesis of CS-D/E decreases the endogenous uptake of Otx2 by PV-cells in the mouse visual cortex, giving further weight to the idea that these complex sugars are true binding sites for Otx2 [56]. Most importantly, we observed that blocking Otx2 import in the adult and thus decreasing its concentration within PV-cells has a strong effect on PV expression and PNN assembly as if PV-cells were rejuvenated [55]. The latter biochemical "rejuvenating" effect of blocking Otx2 capture by PV-cells is associated with the reopening of a period of plasticity. This was shown in two models. In a first model we closed one eye in mice previously infused the RK-peptide, thus with a diminished Otx2 content in PV-cells. Normally this does not lead to a decrease of visual acuity since CP for binocular vision is closed in the adult. However, following RK-peptide infusion, eye closure led to a loss in visual acuity demonstrating a transient recovery of plasticity. More interestingly, infusing the RK-peptide in amblyopic mice restored normal visual acuity, thus curing the mice from a neurodevelopmental disease [55]. 

The origin of Otx2 will not be fully discussed in this review. We have shown that biotinylated Otx2 injected in the eye is transported to PV-cells thanks to the passage of several synapses, but they are probably other sources [54, 57]. One putative source is the choroid plexus that expresses Otx2 throughout life [54, 57] and our preliminary experiments do suggest a contribution of this structure to PV-cell Otx2 content. The latter possibility is of interest as Otx2 is not present only in PV-cells from the visual cortex but throughout the cerebral cortex, suggesting that the control of plasticity by this HP, demonstrated in the visual cortex, could be of a more general value. This will be explored in the years to come within the context of a function of PV-cell number and maturation in psychiatric diseases [48]. At this point the results summarized above allow me to propose a two-step model in CP opening, closure and reopening in the cerebral cortex. This model illustrated in (Fig. **10B**) stipulates that Otx2 progressively captured by PV-cells reaches a first concentration threshold that opens plasticity and, later on, a second one that closes plasticity. Maintaining Otx2 above the second threshold keeps the circuits in a non-plastic state with a low E/I balance but going backward can reduce inhibition and reopen plasticity. In this adapted version of Wolpert's "French Flag" model [58] where space is replaced by time, plasticity is a default state that has to be repressed by the continuous internalization of Otx2.

## THERAPEUTIC PROTEINS

To shift gear temporarily, I will describe the use of HPs as potential therapeutic proteins. The cell penetrating peptide Penetratin, derived from the HD third helix and used to deliver hydrophilic agents into the cell interior has opened the field of transduction peptides that has been reviewed several times [15, 18]. In contrast the use of full-length HPs as therapeutic tools has not attracted the attention that it may deserve. The first use of a HP in a "therapeutic context" was reported in 2003 with the ability of HoxB4 to enhance the proliferation of hematopoietic precursors [59]. In the same context of progenitor proliferation or differentiation one can cite the recent work on Pdx1 that drives human Embryonic stem cells into insulin producing cells [60]. 

In our own laboratory, we have analyzed the use of Otx2 in RGC survival in a mouse model of glaucoma. We showed that Otx2 injected into the eye cup and internalized by the cells saves RGCs and that this protection preserves visual acuity measured in the optomotor test [61]. Studies *in vitro* allowed us to establish that the protein acts at low concentrations (below 3 nM) and that its activity requires its internalization. Similarly, Engrailed survival effect was analyzed for the mesencephalic dopaminergic (mDA) neurons. mDA neurons from the Substantia Nigra (SN) and Ventral Tegmental Area (VTA) that die in Parkinson Disease (PD) express En1 and En2 in the adult and experience progressive mDA cell death in a *En1+/-* mouse line [62]. As in PD, neuronal death is more accentuated in the SN than in the VTA with 40 and 20% death, respectively, after one year and leads to the motor and non-motor behavior deficits observed in the human disease. The two death rates correspond to what is normally observed in PD, leading us to speculate that En1 might be in the "PD pathway". En1 or En2 infused into the brain at the level of the SN was internalized by the cells and blocked cell death [62].

This was not so surprising as it consisted in rescuing a genetic loss of function by a pharmacological gain of function. This is why the effect of En1 was further tested in 3 PD classical models: 6-hydroxi-dopamine, MPTP and alpha-A30P-synuclein toxicity. In the 3 models, En1 protected the mice both promoting mDA neuron survival and increasing the amount of dopamine synthesized per cell [44]. This protection was also followed at the behavior level. Most drugs that affect mDA survival act at the level of mitochondrial complex I [63, 64]. Since we had observed that En1 and En2 enhance the translation of Ndufs1 and Ndufs3, we verified if blocking Ndufs1 translation with the help of siRNAs would hamper En1 pro-survival activity and found that this is indeed the case. Accordingly we observed that the amount of Ndufs1 and Ndufs3 is reduced by 30% in the mDA neurons of one year old *En1+/-* mice. 

## PERSPECTIVES

The finding that HPs are signaling molecules raises many issues that will need to be addressed in the future and cannot all be listed. Many of them may still be to identify as it is impossible to embrace the consequences of having 200 to 300 novel signaling molecules to be added to the repertoire of established signaling entities. However one can tentatively discuss some cell biological, developmental and physiological issues. 

At the cell biological level, the precise mechanisms of secretion and internalization still need to be dissected. Secretion is not blocked by brefeldin A, thus does not involve the ER-Golgi pathway, and they are presently more hypotheses than solid data to explain non-canonical secretion. This holds true for HPs but also for many other proteins secreted in the absence of a signal peptide, like TAT transcription factor, IL1ß, thioreduxin and a few others. Moreover the regulation of secretion and why a passage through the nucleus is seemingly necessary needs to be explored [21]. Internalization is also quite mysterious [12, 13]. Mutations were identified in the third helix of the HD (Penetratin) that block internalization of this 16 amino-acid long peptide and the same mutations also block HP internalization suggesting shared mechanisms. HPs can be captured in absence of endocytosis and, even if endocytosis takes place in some conditions or for some HPs, the endosomal membrane must also be transduced to permit access to the cytoplasm and nucleus. The best explanation, based on phosphorus NMR [65], is a HP-induced transient disturbance of the lipid bilayer possibly followed by rapid repair, either spontaneous or enzymatic as is the case for muscle fibers [66].

**Fig. (11):**
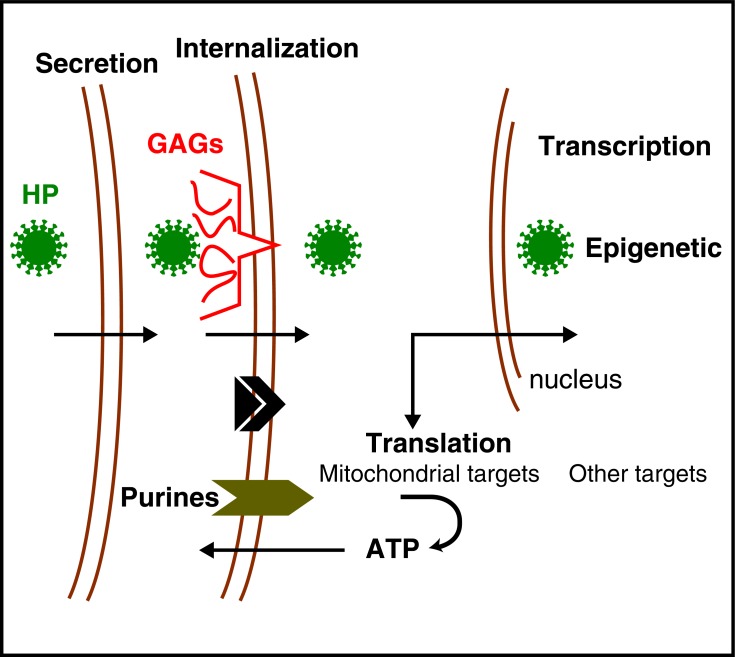
Cell biological agenda for HP signalling. Following secretion through a still not fully understood pathway, HPs must recognize their target cells and complex sugars of the glycosaminoglycan (GAG) family are probably involved raising the "sugar code" issue. Internalization is not well understood and probably involves a local an transient destabilization of the membrane followed by rapid repair. Within the cells, HPs have translational and transcriptional targets that need to be identified and an epigenetic activity that remains to be fully characterized. HP signaling in the best known cases synergizes with other signaling pathways that might vary depending on the situation (type of HP, type of target cell, age, etc). Finally, in the case of Engrailed and Otx2, internalization is followed by the translation of mitochondrial mRNAs and ATP synthesis and secretion and it is not known whether this can be generalized to all other transducing HPs.

Still at the cell biological level, one has to identify the HP receptors or binding sites. In the case of Otx2 they are provided by GAGs that dictate the specificity of recognition [55, 56]. Sequences homologous to the GAG-binding domain identified in Otx2 are present in most HPs, suggesting the existence of a sugar code for HP recognition. Confirming the existence of such a code and cracking it is indeed high on the priority list. This list can be completed with the questions illustrated in (Fig. **11**). First, is the co-signaling concept and the regulation of ATP synthesis a general property of signaling HPs? Second what are the non-cell autonomous targets? This includes the two levels of transcription and translation regulation. Third, on the basis of the long lasting effect of En1 treatment on mice behavior (unpublished data), it is possible that HPs exert their activity also at the epigenetic level. Based on preliminary experiments, we speculate that some HPs, at least, are active in the regulation of CpG methylation, histone modifications and, more generally, in the regulation of chromatin structure and genome stability. Investigating the latter points is on our agenda.

**Fig. (12):**
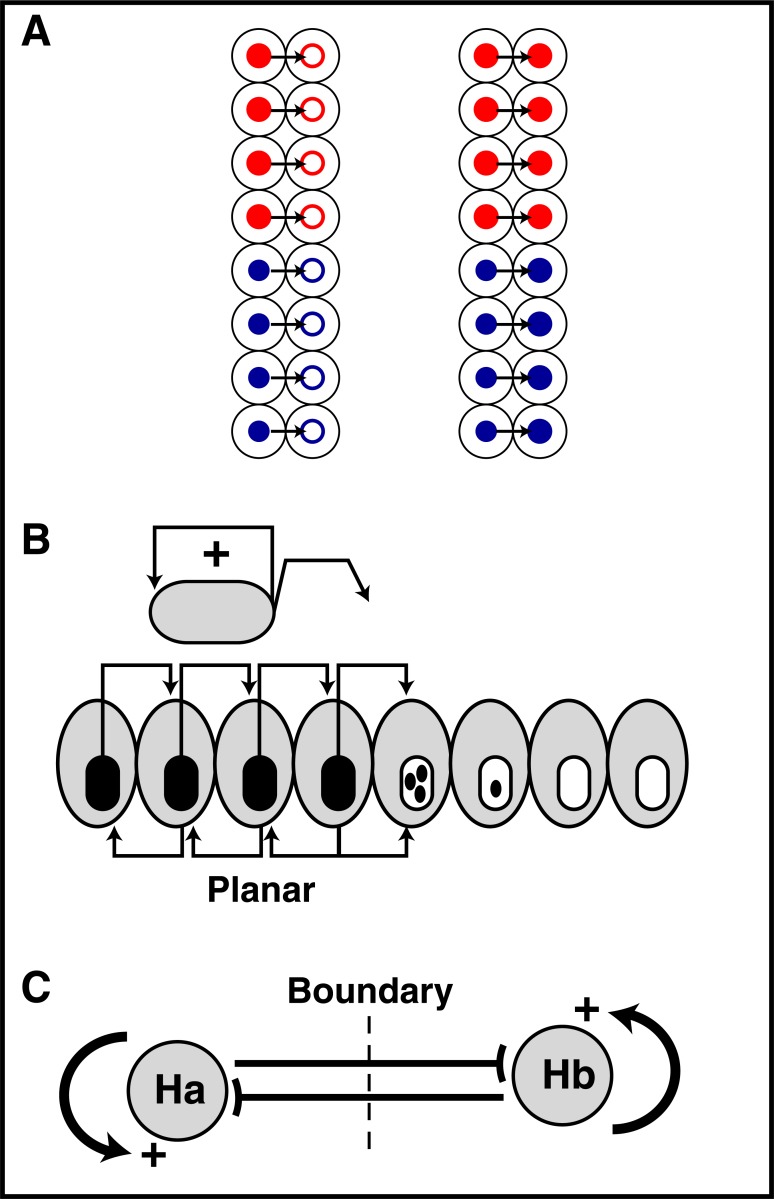
Early developmental role of HP transfer: a hypothesis. **A**. Vertical induction of positional information between two layers, for example the mesoderm and the neurectoderm. In this scheme HPs expressed in one layer are transported into an abutting layer and induce compartmentalization. **B**. Planar induction whereby the protein induces its own expression in abutting cells and thus spreads and increases the size of its own territory. **C**. A boundary can form where two "infectious" HPs with self-activating and reciprocal inhibitory activities meet.

Developmental issues are also numerous. In a 2003 review we had proposed a role of HP transfer in guidance and, more generally, in the transfer of positional information (e.g. between mesoderm and neuro-ectoderm) and in compartmentalization of the cortex [14]. Guidance was studied since it is simpler to work at late developmental stages than at very early developmental stages. We however used the zebrafish model to express an extracellular anti-Pax6 single-chain antibody *in vivo* or infuse a polyclonal anti-Pax6 antibody in the blastula and observed that this leads to a reduced eye field [67]. These data were interpreted as a support to the idea that HPs are infectious proteins (Fig. **12**). Very often, at either side of a boundary, HPs with self-activating and reciprocal inhibitory activities are expressed, and modifying the expression of one of the two HPs also modifies boundary position. This is illustrated, among many other cases, by the role of the Otx2/Gbx2 balance in the positioning of the midbrain-hindbrain boundary [68, 69]. This has led us to propose a function of non-cell autonomous HP activity in the compartmentalization of the neuroepithelium illustrated in (Fig. **12**) [70]. Although provocative and exciting this hypothesis is difficult to test due to the very early stages at which these events take place and also because the transfer sequences are in the HD, forbidding to produce mutants in which only the non-cell autonomous activities would be lost. This is why we have now developed mouse lines in which extracellular antibody expression will be induced at desired time and place. This will allow us to study the consequences of blocking HP extracellular activity at very early developmental periods.
